# Effects of Cancer Treatment on the Education and Cognition of South Florida Pediatric Cancer Survivors

**DOI:** 10.7759/cureus.37001

**Published:** 2023-04-01

**Authors:** Jessica R Fine, Tanvi Bafna, Sarah C Griffith, Justine M Ransdell, Julio C Barredo, Derek M Isrow

**Affiliations:** 1 Department of Radiation Oncology, University of Miami Miller School of Medicine, Miami, USA; 2 Department of Pediatrics, University of Miami Sylvester Comprehensive Cancer Center, Miami, USA; 3 Department of Radiation Oncology, University of Miami Sylvester Comprehensive Cancer Center, Miami, USA

**Keywords:** pediatric oncology, disparities, education, cancer survivorship, pediatrics

## Abstract

Purpose

As pediatric cancer survival rates have exponentially increased in the past decade, with the vast majority surviving five years or more, the long-term impacts of treatment on the quality of survivorship must be explored. This study examines the effects of pediatric cancer treatment regimens on education outcomes among a demographically diverse regional population. The primary objective is to identify potential factors that may impact the educational and cognitive quality of life in this population.

Methods

Four hundred sixty-eight pediatric oncology patients diagnosed at age <20 between January 1990 - August 2019 and treated for cancer with radiation therapy at a large public or a multi-center private hospital in South Florida were identified. A novel survey available in English and Spanish was electronically distributed at least three times to each patient from August 2020 - July 2021 via email, phone call, and text message. Variables relating to demographics, treatment, cognitive impairment, and school re-entry were collected through the survey and electronic medical record review. Descriptive statistical analysis was performed.

Results

Of the patients, 10.5% responded to the survey (26 male, 21 female, two unidentified sex). The mean age was 8.9 years old (range 0-20) at diagnosis, 24.0 years old (range 8-39) at the time of survey completion, and 55.1% self-identified as Hispanic. Nearly one-quarter of respondents (22.4%) were unable to correctly identify the treatment modalities they received; Hispanic self-identifying patients were 1.75 times more likely than non-Hispanic patients to incorrectly report the treatment modalities received. One-quarter (26.5%) of respondents reported long-term cognitive deficits post-treatment, of which, over three-quarters (76.9%) identified as Hispanic.

Conclusion

This study illuminates patients' perspectives on their long-term cognitive impacts after pediatric cancer treatment. Given the diverse study population, ethnic disparities in post-treatment survivorship were explored. A substantial subset of Hispanic participants was unable to correctly identify their treatment regimen, and a disproportionately large group of Hispanic patients experienced cognitive long-term cognitive deficits, suggesting that ethnic disparities play a critical role in post-treatment survivorship. Further research on prioritizing educational intervention during and after treatment is essential to improving both the quality and equity of survivorship among pediatric oncology patients.

## Introduction

With recent advancements in treatment, 85% of pediatric cancer patients are now surviving five years or more [1]. Given this paradigm shift in survival, research, and societal efforts must also shift focus to maximize the quality of this survivorship. Education is a key measure of long-term survivorship quality as it is crucial to many domains of living and integration in society for survivors of childhood cancer [2]. 

One of the major adverse effects of pediatric cancer treatment is the disruption of normal brain development and subsequent educational achievement. Two-thirds of pediatric oncology patients experience at least one long-term sequelae, including but not exclusive to neurocognitive deficits [3]. Recent research indicates that these long-term effects are partially attributed to the injurious impact of therapeutic interventions such as surgery, chemotherapy, and radiation therapy, in addition to a cancer diagnosis, being an adverse childhood experience [4]. Children with cognitive treatment effects are more likely to have lower academic achievement as well as difficulty with employment, living independently, and developing social relations [5]. In addition to effects attributed to the medical treatment of their disease, pediatric cancer patients often experience prolonged school absences. This can hinder overall education attainment as they are often missing school during their vital learning periods. A report from the Childhood Cancer Survivor Study on educational attainment in long-term survivors of childhood cancer notes that all survivors, regardless of their type of treatment, were significantly less likely to finish high school when compared to their siblings [6]. Further research is necessary to circumvent the adverse cognitive outcomes of treatment and to enhance educational outcomes among survivors of pediatric cancer. 

This study examines the effects of cancer treatment on educational outcomes and learning ability in a uniquely diverse regional population. Approximately 46% of the population in South Florida (including Miami-Dade, Broward, and Palm-Beach counties) identify as Hispanic, 22% as Black non-Hispanic, and 25% as white non-Hispanic [7]. This demographic diversity not only reflects the changing national population but also allows for further evaluation of the role of demographics in survivorship [8]. 

Preliminary findings of this study were presented as a poster presentation at the virtual International Cancer and Education Conference (ICEC) on October 15, 2021, and the virtual University of Miami American Physician Scientist Association (APSA) Symposium on December 4, 2021. In addition, an oral presentation was given at the Eastern-Atlantic Student Research Forum (ESRF) in Miami, Florida, on February 13, 2022.

## Materials and methods

This study was approved by the University of Miami Institutional Review Board. Patients were identified by having received radiation therapy as a part of their cancer treatment. All study patients were diagnosed with cancer between January 1990 and August 2019 at an age less than or equal to 20 years old. All participants received treatment at the University of Miami Sylvester Comprehensive Cancer Center (SCCC) or Jackson Health System (JMH). The majority of patients were residents of South Florida, primarily Miami-Dade County or Broward County. Four hundred sixty-eight patients meeting the inclusion criteria were identified. Data was collected via a retrospective review of electronic medical records (EMR) and patient completion of a survey.

A retrospective review of each patient's EMR was conducted by two individual reviewers. The sociodemographic variables collected included sex, race, and ethnicity. Sex was defined as male or female. Race categories included white, Black, other, and unknown. Ethnicity was defined as Hispanic, non-Hispanic, or unknown. The clinical variables collected included diagnosis type and treatment modalities. Individual diagnoses were grouped into categories adapted from the International Classification of Childhood Cancer, which included leukemias, lymphomas, gliomas, sarcomas, pediatric central nervous system (CNS) tumors, and other solid cancers. Treatment modalities were defined as no treatment, surgery only, radiation only, chemotherapy only, surgery and radiation, radiation and chemotherapy, surgery and chemotherapy, and surgery, chemotherapy, and radiation. Surgery, radiation, and chemotherapy variables were defined as either yes, no, or unknown based on whether that particular treatment was received. Survey respondent data was subsequently linked to corresponding EMR data. Of note, two survey respondents were unable to be linked to their EMR due to incomplete responses to identifying information in the survey. 

A novel survey was designed on the survey platform Qualtrics (Qualtrics, Seattle, Washington). Questions were written from knowledge gaps and future directions identified upon a literature review of education in pediatric cancer survivorship [9-11]. The survey was translated into Spanish by a certified medical interpreter. The survey was distributed between August 2020 and July 2021 in both English and Spanish to all patients in the study group. Each patient was sent the survey up to three times, at least one time by each of the following modalities: email, phone call, and text message. After three attempted contacts, if the patient did not respond, they were not contacted again.

The sociodemographic variables collected in the survey included sex, race, ethnicity, first language learned, and language most frequently spoken at home. The clinical data collected included diagnosis and treatment modalities (as defined above). Education information elicited included the duration of school absence and whether the school attended after treatment was prepared to handle the educational needs of childhood cancer survivors. Five-point Likert scales were used to identify qualitative variables, including the stress levels experienced by survivors and their families during the school re-entry process. Cognitive functional status was assessed via patient perceptions of any cognitive deficits experienced after treatment and at the time of survey completion. Quality of communication with the cancer care team was explored via Likert scales. Communication was also assessed via patient identification of whether counseling on potential cognitive deficits that could result from cancer treatment was performed. 

The data analysis was performed with SAS software (SAS Institute Inc., Cary, North Carolina) and Microsoft Excel® 2022 (Microsoft Corporation, Redmond, Washington).

## Results

Survey respondent demographic results

Of the 468 patients contacted, 49 (10.5%) responded to the survey. The demographic characteristics of the study sample are given in Table 1. The survey respondents consisted of 26 males, 21 females, and two who did not identify their sex. The majority of respondents self-identified as Hispanic (55.1%). Others self-identified as white (49.0%), Black (16.3%), other (28.6%), and 6.1% chose not to identify their race. More than half (59.2%) reported English as their first language learned. Most respondents, 71.4%, primarily spoke English at home, while 26.5% spoke primarily Spanish at home, and 2.0% spoke another language at home (Table 2). On average, respondents were 8.9 years old at diagnosis, with no variation by ethnicity. The average age of respondents upon survey completion was 24.0 years old (range 8.8-39.4). The average duration between the patient's diagnosis and survey completion was 14.1 years (Table 3). 

**Table 1:** Baseline demographics

Variable	Number (N)	%
All patients	49	100.0
Sex		
Male	26	53.1
Female	21	42.8
Unknown	2	4.1
Race		
White	24	49.0
Black	8	16.3
Other	14	28.6
Unknown	3	6.1
Ethnicity		
Non-Hispanic	22	44.9
Hispanic	27	55.1
Diagnosis type		
Leukemias	7	14.9
Acute lymphocytic leukemia (ALL)	5	10.6
Acute myeloid leukemia (AML)	2	4.3
Central nervous system (CNS) tumors	6	2.8
Medulloblastoma	2	4.3
Primitive neuroectodermal tumor	1	2.1
Germinoma	3	6.4
Sarcomas	10	21.3
Ewing sarcoma	2	4.3
Rhabdomyosarcoma	5	10.6
Sarcoma	1	2.1
Soft tissue sarcoma	1	2.1
Alveolar soft part sarcoma	1	2.1
Gliomas	2	4.3
Glioma	2	4.3
Lymphomas	9	19.2
Hodgkin lymphoma	6	12.8
Non-Hodgkin lymphoma	2	4.3
Lymphoma	1	2.1
Neuroblastoma and peripheral nervous cell tumors	2	4.3
Neuroblastoma	2	4.3
Retinoblastomas	5	10.6
Retinoblastoma	5	10.6
Renal tumors	4	8.5
Wilms tumor	4	8.5
Other	2	4.3
Nasopharyngeal cancer	1	2.1
Adenoid cystic carcinoma	1	2.1

**Table 2:** Communication with cancer care team by first language

Variable	All Patients, N=49, N (%)	English, N=29, N (%)	Spanish, N=19, N (%)	Other, N=1, N (%)
Language at home				
English	35 (71.4)	27 (93.1)	8 (42.1)	0 (0.0)
Spanish	13 (26.5)	2 (6.9)	11 (57.9)	0 (0.0)
Other	1 (2.0)	0 (0.0)	0 (0.0)	1 (100.0)
Family ability to communicate with care team				
Excellent	28 (57.1)	17 (58.6)	10 (52.6)	1 (100.0)
Good	9 (18.4)	4 (13.8)	5 (26.3)	0 (0.0)
Ok	1 (2.0)	1 (3.5)	0 (0.0)	0 (0.0)
Poor	3 (6.1)	2 (6.9)	1 (5.3)	0 (0.0)
Terrible	0 (0.0)	0 (0.0)	0 (0.0)	0 (0.0)
Unknown	8 (16.3)	5 (17.2)	3 (15.8)	0 (0.0)
Personal ability to communicate with care team				
Excellent	24 (49.0)	13 (44.8)	10 (52.6)	1(100.0)
Good	11 (22.5)	7 (24.1)	4 (21.1)	0 (0.0)
Ok	4 (8.2)	3 (10.3)	1 (5.3)	0 (0.0)
Poor	1 (2.0)	1 (3.5)	0 (0.0)	0 (0.0)
Terrible	1 (2.0)	0 (0.0)	1 (5.3)	0 (0.0)
Unknown	8 (16.3)	5 (17.2)	3 (15.8)	0 (0.0)

**Table 3:** Age at diagnosis and survey completion by ethnicity

Variable		Total (N=49)	Hispanic (N=27)	Non-Hispanic (N=22)
Age at diagnosis (years)	Mean	8.9	8.8	9.0
	SD	5.6	5.4	6.2
	Median	9.0	8.0	10.5
	Min	0.0	1.0	0.0
	Max	20.0	20.0	19.0
Age at survey completion (years)	Mean	24.0	24.3	23.7
	SD	6.8	6.2	7.7
	Median	24.8	23.8	25.9
	Min	8.8	15.5	8.8
	Max	39.4	39.4	34.5
Time elapsed between date of diagnosis and survey completion (years)	Mean	14.1	13.7	14.6
	SD	6.1	5.7	6.8
	Median	13.2	13.2	13.2
	Min	5.6	5.6	5.8
	Max	30.7	28.3	30.7

Survey respondent cancer diagnosis and treatment results 

The largest diagnostic group represented by survey respondents was sarcomas which included 10 patients (21.3%). All survey participants (100.0%) received radiation therapy during their cancer treatment; 23 patients (46.9%) received whole brain radiation therapy (WBRT), and 22 patients (44.9%) received extracranial radiation therapy (Table 4). 

**Table 4:** Treatment regimen and survey concordance with EMR by ethnicity EMR - electronic medical record

Variable	All patients, N=49, N (%)	Hispanic, N=27, N (%)	Non-Hispanic, N=22, N (%)
Treatment regimen			
Radiation alone	2 (4.1)	2 (7.4)	0 (0.0)
Chemotherapy and radiation	18 (36.7)	12 (44.4)	6 (27.3)
Chemotherapy, radiation, and surgery	25 (51.0)	12 (44.4)	13 (59.1)
Radiation and surgery	2 (4.1)	1 (3.7)	1 (4.5)
Unknown	2 (4.1)	0 (0.0)	2 (9.1)
Whole brain radiation therapy			
Yes	23 (46.9)	15 (55.6)	8 (36.4)
No	22 (44.9)	12 (44.4)	10 (45.5)
Unknown	4 (8.2)	0 (0.0)	4 (18.2)
Survey response matching EMR record			
Yes	31 (63.3)	18 (66.7)	13 (59.1)
No	11 (22.4)	7 (25.9)	4 (18.2)

Concordance between patient-reported treatment modalities and treatment modalities as documented in the EMR revealed that, of those who identified their treatment plans on the survey, nearly one quarter (22.4%) were unable to correctly identify the combination of treatment modalities they received according to EMR documentation. Of patients whose reported treatment was discordant with the EMR, 63.6% were Hispanic, and 36.4% were non-Hispanic (Table 4).

Education after diagnosis and return to school 

In sum, 40.8% of total respondents reported missing school for at least six months. Patients who received WBRT (46.9%) reported a longer period of absence from school during their cancer treatment (26.0% reported absence >1 year) than those who received extracranial radiation (9.1% reported absence >1 year) (Table 5).  

**Table 5:** School absence by WBRT status WBRT - whole brain radiation therapy

Total school absence during Tx	All patients, N=49, N (%)	WBRT, N=23, N (%)	No WBRT, N=22, N (%)	Unknown, N=4, N (%)
< 6 months	11 (22.4)	4 (17.4)	6 (27.3)	1 (25.0)
6-12 months	15 (30.6)	5 (21.7)	9 (40.9)	1 (25.0)
1-2 years	1 (2.0)	1 (4.3)	0 (0.0)	0 (0.0)
> 2 years	7 (14.3)	5 (21.7)	2 (9.1)	0 (0.0)
Unknown	15 (30.6)	8 (34.8)	5 (22.7)	2 (50.0)

**Figure 1:**
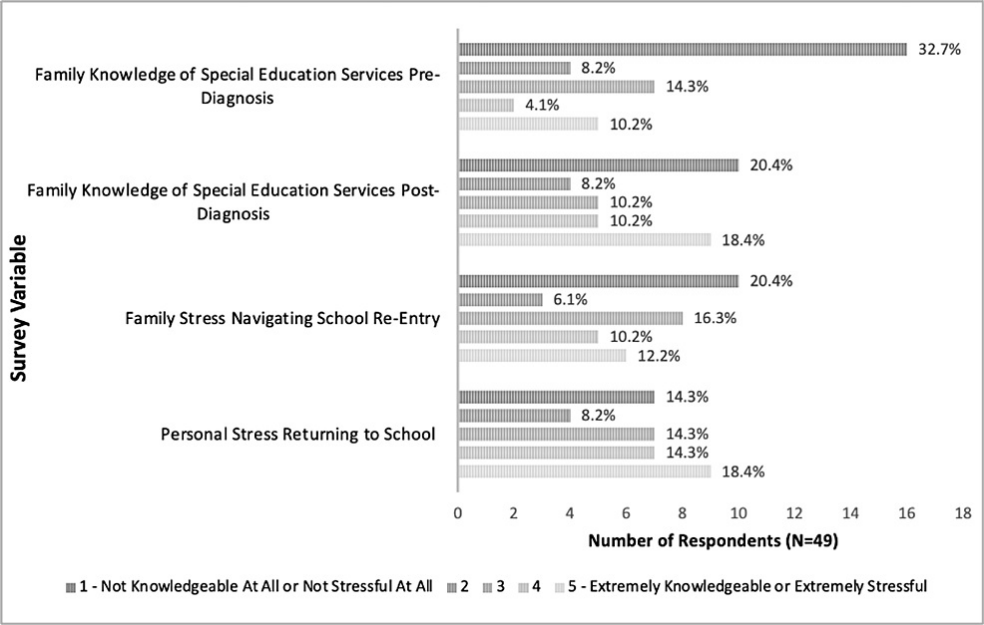
Family knowledge of special education services and return to school stress A Likert scale from 1-5 was used to respond to the shown survey questions wherein 1 = not knowledgeable at all or not stressful at all and 5 = extremely knowledgeable or extremely stressful. The remaining percentage of respondents not illustrated did not respond to the specific survey variable.

On the five-point Likert scale, 32.7% of respondents rated their stress upon returning to school as a four or five, with five signifying an "extremely stressful" process (Figure 1). Only 6.1% of respondents indicated that their school was equipped to handle the educational needs of cancer survivors.

Cognitive deficits 

Approximately one-third (34.7%) of respondents reported experiencing cognitive deficits after receiving treatment; of these, nearly three-quarters (70.6%) identified as Hispanic. One-quarter (26.5%) of total respondents indicated that because of their cancer treatment, they continued to have learning difficulties at the time of survey completion, of which over three-quarters (76.9%) identified as Hispanic and 23.1% as non-Hispanic (Table 6). Some of the barriers that patients reported facing upon their re-entry to school included financial burden, an increased effort to concentrate and retain information, memory loss, diminished reading ability, difficulty walking, and physical defects from treatment.

**Table 6:** Cognitive deficits post-treatment and care team communication of side effects by ethnicity

Variable	All patients, N=49, N (%)	Non-Hispanic, N=22, N (%)	Hispanic, N=27, N (%)
Self-reported learning deficit			
Yes	17 (34.7)	5 (22.7)	12 (44.4)
No	19 (38.8)	11 (50.0)	8 (29.6)
Unknown	13 (26.5)	6 (27.3)	7 (25.9)
Continued Impact on Ability to Learn			
Yes	13 (26.5)	3 (13.6)	10 (37.0)
No	22 (44.9)	13 (59.1)	9 (33.3)
Unknown	14 (28.6)	6 (27.3)	8 (29.6)
Notified of risk of cognitive deficits by care team			
Yes	21 (42.9)	6 (27.3)	15 (55.6)
No	6 (12.2)	3 (13.6)	3 (11.1)
I don't remember	14 (28.6)	9 (40.9)	5 (18.5)
Unknown	8 (16.3)	4 (18.2)	4 (14.8)
Notified of side effects by care team			
Yes	32 (65.3)	12 (54.5)	20 (74.1)
No	3 (6.1)	3 (13.6)	0 (0.0)
I don't remember	6 (12.2)	3 (13.6)	3 (11.1)
Unknown	8 (16.3)	4 (18.2)	4 (14.8)

Communication with the care team 

Approximately half of the respondents (49.0%) reported that they personally had an excellent ability to communicate with their care team, while 57.1% of respondents indicated that their families had an excellent ability to communicate with the care team (Table 2). Less than half of patients (42.9%) reported that they were notified of potential cognitive deficits as a result of treatment.

## Discussion

Survey respondent demographics, diagnosis, and discordance of treatment

The study population is specific to pediatric patients that received radiation therapy as part of their cancer treatment regimen. The racial, ethnic, and linguistic composition of the survey respondent population reflects the rapidly changing demographic diversity both regionally in South Florida and nationally. Representing these growing communities corroborates the generalizability of the educational disparities experienced by the survey population. Not only does this study enable the oncology community to affect change in populations typically underrepresented in medical research, but it also enables the identification of gaps in post-treatment quality of life to ameliorate the inequities experienced by these groups.

Pediatric cancer patients may be at increased risk for poor educational outcomes due to incomplete or misunderstanding of their diagnosis and treatment plan. Given that only 63.3% of study participants were able to correctly report the treatment modalities they received, it is evident that treatment discordance is a prominent shortcoming among patients. This may be due in part to children often being too young to understand or remember the details of their disease, as well as intentional parental shielding [12]. Furthermore, it was found that Hispanic patients were 1.75 times more likely to incorrectly report the treatment modalities they received (lower concordance with EMR records) than their non-Hispanic counterparts. This suggests an ethnic disparity within the knowledge of childhood cancer survivors regarding their past treatment, potentially increasing the risk for future development of treatment-related complications.

Decreased awareness of treatment is a critical disparity to consider because it can impair the ability of pediatric cancer patients to identify and predict the therapy-related adverse outcomes they may endure. Several factors, including lower education level, longer time elapsed from diagnosis, and nonwhite race, are significantly associated with future personal health risks among survivors of childhood cancer [13]. Additionally, disparities in communication at the time of treatment can further precipitate the evolvement of unmet emotional needs, heightened uncertainty, and lack of insight into short and long-term cognitive side effects [14]. To promote equity in patients' understanding of their medical care, physicians should emphasize patient education among patient populations at increased vulnerability.

Education after diagnosis and return to school 

A unique commonality of the study population was that all respondents received radiation as part of their treatment plan; furthermore, the radiation sites (WBRT versus extracranial) were equally represented among the respondents. The site of radiation delivery is a valuable marker that may facilitate a better understanding of the toxicities associated with receiving general radiation compared to receiving direct-brain radiation. This was a particular area of interest because it is known that the developing brain of pediatric patients is sensitive to chemotherapy and cranial radiation, resulting in potential structural changes and memory impairment [15-16]. According to a report from the Children's Oncology Group, children treated with WBRT are noted to have greater declines in neurocognitive function with decreases in IQ as much as 15 to 25 points [3]. Additionally, patients who received WBRT were three times more likely to miss more than one full year of school than their counterparts who received radiation to any other organ system. Similar results were reproduced in the conduction of this study, with three times as many WBRT participants missing one year of school or greater than those who did not receive WBRT. This may put children who received WBRT at greater risk for falling behind not only in their educational development but also in social development due to decreased peer interaction, ultimately leading to poorer outcomes later in life [17, 18]. In this study, participants were asked to share barriers they faced upon returning to school. Those who received WBRT specifically reported difficulties in concentrating and retaining information, reading and writing at grade level, and socializing after a period of isolation. Based on the intersection of these findings, pediatric patients who receive WBRT may be more vulnerable to adverse educational outcomes relating to prolonged school absence than those receiving extracranial radiation. Clinicians should proactively focus on the WBRT population when communicating the long-term neurocognitive risks and creating a plan to achieve non-inferior educational attainment.

Cognitive deficits 

The presentation of cognitive deficits in children treated with radiation therapy varies widely but often involves executive functioning, including attention, memory, and processing speed [19-20]. Over one-third of respondents suffered cognitive deficits post-treatment; however, just 6.1% of total respondents indicated that their school provided services to alleviate this. More than one-quarter of respondents endorsed having persistent cognitive deficits at the time of survey completion. Given that the average respondent age was 24 years old, this study was able to elicit the unique perspective of young adults reflecting on how their own childhood illness has impacted their cognitive and educational transition into adulthood. To improve outcomes, a multidisciplinary team for guidance through the school re-entry process and arrangement of regular neurocognitive follow-up should be the standard of care for all pediatric cancer patients. These services are essential as they promote positive adjustment among survivors and improve collaboration between the community and the healthcare team [21].

Communication with the care team 

This study revealed deficits in communication that may have influenced the long-term outcomes of pediatric cancer survivors. Given that 12.2% of survey respondents indicated that communication with their care team was ok, poor, or terrible, there is room for improvement in the quality and clarity of interactions between patients and providers. Potential factors that may have influenced inferior communication with the healthcare team include language differences or suboptimal establishment of rapport between patient and clinician, among other factors. 

Remarkably, less than half of the respondents recall being notified of potential cognitive deficits they might experience during treatment. Literature on cancer care communication to date has largely arisen from the psychosocial support domain, including psychologists, psychiatrists, and social workers, rather than from the primary oncology team [14]. Although access to these services is an essential component of delivering multidimensional care, it is imperative that all providers communicate clearly and empathetically during every encounter to ensure fidelity of the information and maintain rapport with the patient.

Strengths and limitations 

The paramount strength of this study was the unique ethnic diversity of the study population. It is not only representative of the South Florida region but also future national demographics, given the projected growth of the Hispanic population [22]. This strengthens the generalizability of the results by elucidating the disparities experienced by minorities in a national population more diverse than ever recorded [23-24]. Moreover, the findings of the study enable providers to identify groups on which to focus targeted interventions. The diversity in this region also provided an adequate context to examine the role of language in access to care and quality of care. In addition to the sociodemographic generalizability, the study population's diagnostic groupings are also reflective of the pediatric oncology population at large [25]. Another strength was the utilization of an extensive regional pediatric database of patients who were diagnosed as early as 1990 and received radiation therapy as part of their oncological therapeutic regimen. The longevity of this database enabled the collection of long-term education and cognition data, thus greatly expanding the potential to assess the post-treatment deficits specific to radiation therapy. These strengths ultimately enhance the interpretation, relevance, and reliability of the study findings.

Nevertheless, certain limitations must be considered when interpreting the findings of this study. One limitation is the lack of external validity of the clinical questionnaire that was created for this study. However, an extensive literature review was utilized in the curation of the survey, with additional internal review by pediatric oncologists. While this tool has not been previously validated, the study team found it necessary to generate a novel survey to target knowledge gaps identified in previous publications. Other salient limitations pertain to sample size and survey response. Of those eligible to participate, 10.5% completed the survey for a total sample size of 49. A barrier to successfully contacting patients was inactive contact information in the EMR, as many had completed treatment over a decade since the survey was distributed. This decreased the number of patients successfully reached via phone, email, or text message and may have hindered the response rate. With a patient population just shy of 50, the generalizability of the results may be limited due to the increased risk for bias that is introduced by low survey response rates [26]. To encourage patient completion of the survey, some questions were not required to be answered for submission. As a result, some respondents did not complete the survey in full. This led to varied degrees of missing data for certain variables. Some respondents may have been sensitive to certain questions or subject matter, increasing the potential for nonresponse bias [27]. Thus, survey questions that have variable completion rates may be less generalizable to the greater community.

Furthermore, the length of time over which diagnoses spanned was also variable. While the majority of respondents were treated in the last decade, others were treated two or three decades ago. Interpretation of results may be limited by recall bias as patients who completed treatment more recently may remember more details of their care than those who were further removed [27]. Additionally, measures of cognitive deficit may have been limited by the age at which participants were treated for their cancer. For example, it is unclear whether patients diagnosed and irradiated at age 18-20 would experience similar adverse cognitive effects as younger children whose brains are in critical stages of development. It is also important to consider that patients undergoing whole brain radiation may have had underlying baseline cognitive and educational deficits, which may make it difficult to ascertain how much of the cognitive deficits were due to treatment.

Future directions 

This study has identified groups most vulnerable to adverse cognitive outcomes following cancer treatment, specifically those identifying as Hispanic or treated with WBRT. Future efforts to design and implement targeted interventions should focus on these groups to promote equitable outcomes among all childhood cancer patients. More broadly, further research is necessary to analyze the specific cognitive impairments and challenges present among this population of childhood cancer survivors following treatment with radiation. Learning should also be increasingly encouraged and integrated into pediatric cancer treatment protocol to reduce gaps in schooling and optimize educational potential post-treatment. 

## Conclusions

This study addresses the relationship between cancer treatment and education outcomes in the South Florida pediatric oncology population. Survey respondents were found to experience major gaps in schooling during treatment, difficulties during the back-to-school transition, and perceived deficits in learning ability post-treatment. This study also suggests ethnic disparities may be associated with discordance of treatment awareness and identification of cognitive deficits later in life. Given that many of the South Florida residents identify as Hispanic and that the Hispanic population in the United States continues to grow, these findings are essential to understanding the outcomes of innovative treatments in patient groups typically underrepresented in medical research. Further research is necessary to elucidate specific areas requiring improvement during cancer treatment and care to improve both quality and equity of survivorship amongst pediatric oncology patients.
